# Photo Quiz: Daniel Elmer Salmon, America’s First DVM

**DOI:** 10.3201/eid3104.230702

**Published:** 2025-04

**Authors:** Daniel F.M. Monte, Julia Memrava Cabrera, Fábio P. Sellera

**Affiliations:** North Carolina State University, Raleigh, North Carolina, USA (D.F.M. Monte); São Paulo State University, Jaboticabal, Brazil (J.M. Cabrera); University of São Paulo, São Paulo, Brazil (F.P. Sellera); Universidade Metropolitana de Santos, Santos, Brazil (F.P. Sellera)

**Keywords:** *Salmonella*, bacteria, Bureau of Animal Industry, pathologist, serotypes, typhoid fever, gastroenteritis, public health, food safety

Who is this scientist and what did he accomplish?

Here is a clue: his name is associated with a genus of bacteria that is the causative agent of typhoid fever.

Who is he?

A. Alexander Fleming

B. Theobald Smith

C. Louis Pasteur

D. Daniel Elmer Salmon

E. Robert Koch

Decide first, then see next page for the answer.

This is a photograph of Dr. Daniel Elmer Salmon (July 23, 1850–August 30, 1914) (Figure). Salmon was an American veterinary surgeon and pathologist who authored or co-authored >100 scientific papers and several books and deserves to be designated as one of the three most prominent US veterinarians of the 19th Century. Fellow of the American Association for the Advancement of Science, president of the American Public Health Association and the American Veterinary Medical Association (AVMA), and member of the Washington Academy of Sciences, Salmon made notable contributions to the fields of veterinary medicine and public health, and much of his work corresponded with that of Louis Pasteur in France.

**Figure:**
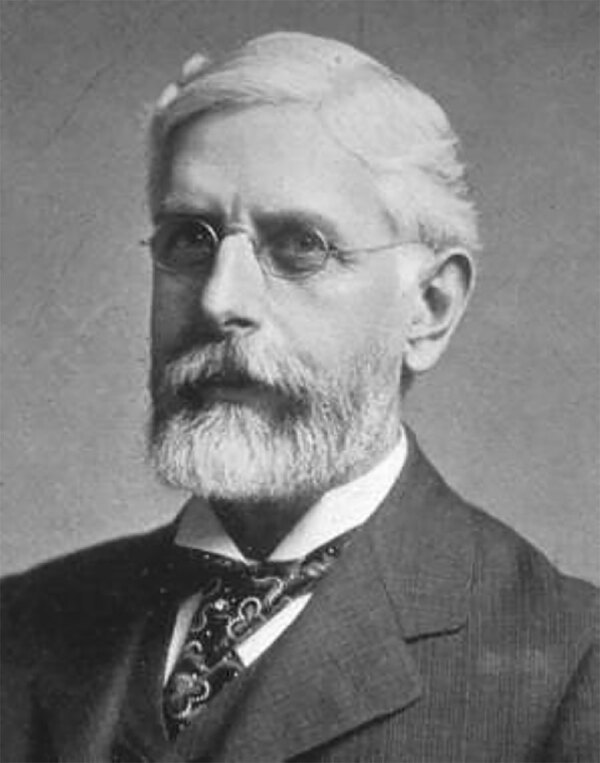
Daniel E. Salmon. Source: Wikipedia (https://es.wikipedia.org/wiki/Daniel_Elmer_Salmon#/media/Archivo:Daniel_Salmon.jpg); public domain image.

Born on July 23, 1850, in Mount Olive, New Jersey, Daniel Elmer Salmon faced adversity early in life, becoming an orphan at the age of 8. After the loss of both parents, he was raised by his second cousin, Aaron Howell Salmon. During that period, he contributed to Aaron’s farm and worked as a clerk in a country store. Salmon pursued his early education at The Mount Olive District School, Chester Institute, and Eastman Business College. In 1872, he married Mary Thompson Corning. After Mary’s death, he remarried to Agnes Christina Dewhurst in 1904.

Salmon studied veterinary medicine at the Bellevue Hospital Medical College in New York, New York, and graduated with a Bachelor of Veterinary Science degree in 1872. He spent the last 6 months of his graduate studies abroad, at the Alfort Veterinary School in Paris, France. In 1876, studying animal diseases at Cornell University, Salmon was awarded the first DVM (Doctor in Veterinary Medicine) degree in the United States. Because of health concerns, Salmon relocated to Asheville, North Carolina, choosing the city for its mild climate at an altitude of 600 meters.

Dr. Salmon became an assistant veterinarian in the United States Department of Agriculture (USDA). During his tenure at USDA as the investigator of animal disease in the southern states, Dr. Salmon played a crucial role in the establishment of the Bureau of Animal Industry (BAI) in 1884. Salmon’s leadership as BAI’s inaugural chief spanned 21 years, from 1884 to 1905, during which time the BAI evolved into a trailblazing institution in the realm of veterinary science. He and his colleagues were instrumental in instituting a comprehensive system for inspecting and quarantining imported animals, as well as implementing the federal meat inspection service.

Dr. Salmon made major contributions to the field of infectious disease research, particularly in livestock. His groundbreaking work included identifying and combating various contagious diseases, such as hog cholera (now known as swine fever), Texas fever (cattle tick fever), and anthrax. Of note, he conducted extensive research on Texas cattle fever (TCF), identifying the causative agent as the protozoan parasite *Babesia bovis*. That research laid the foundation for understanding the disease’s transmission and effects on cattle. Under Salmon’s leadership, the BAI implemented the Texas Fever Tick Eradication Program to control the disease. That comprehensive program aimed to eliminate the tick vector from cattle herds in the southern United States and involved systematic tick removal, quarantine, and treatment of cattle to break the transmission cycle.

In 1879, Salmon played a crucial role in the New York state campaign to eradicate pleuropneumonia in cattle. After that effort, both pleuropneumonia and TCF in cattle were successfully controlled. Recognizing his expertise, the USDA selected Salmon to study the widespread problem of livestock disease in the South, particularly TCF. His investigations and efforts to control those diseases resulted in substantial advancements in veterinary medicine, preventing major outbreaks.

The research on TCF conducted at the BAI stands out as potentially the most influential study ever undertaken at the institute and might be regarded as a pivotal investigation of the 19th Century, shedding light on the capacity of arthropods to transmit diseases. The significance of that study lay in challenging the prevailing skepticism among prominent scientists worldwide, who had dismissed the notion that infectious diseases could be transmitted through insect bites. In a groundbreaking departure from conventional wisdom, Amico Bignami’s seminal article in the Lancet (1896) proposed a revolutionary perspective, asserting that malaria could indeed be transmitted through mosquito bites. That paradigm shift marked a crucial turning point in infectious disease research, challenging preconceived notions and paving the way for a deeper understanding of disease transmission mechanisms.

Salmon’s research extended beyond TCF, encompassing various diseases caused by bacteria. His work made major strides in understanding the etiology and prevention of bacterial diseases, contributing to the overall progress of veterinary medicine. Recognizing his substantial efforts, AVMA acknowledged Salmon as a prominent veterinarian. Beyond his impact on veterinary science, Salmon emerged as key advocate for public health. His influence was particularly evident in the establishment of the Hygienic Laboratory, a foundation later transformed into the National Institutes of Health.

In the realm of infectious disease pathology, Salmon achieved remarkable developments. Of particular note was his validation of the transmissibility of tuberculosis from cattle to humans, affirming the concept of zoonosis, wherein an animal serves as a vector for disease transmission. His contributions also extended to the control of foodborne illnesses, emphasizing the importance of food safety within the farm-to-fork framework. Those insights remain vital to contemporary food authorities and underscore Salmon’s enduring impact on public health and veterinary medicine.

Salmon also investigated the prevention of diseases in sheep, contagious diseases of hogs, and chicken cholera. However, the most notable Salmon achievement was to give his name to the *Salmonella* bacteria genus. Salmon and his assistant Theobald Smith, another prominent microbiologist, recovered a strain of *Salmonella* *choleraesuis* from swine. That finding led to a conundrum that has been a subject of several discussions: who discovered *Salmonella*? The relationship between Salmon and Smith included hearty disputes on the authorship of several research reports, including the most controversial, on *Salmonella*. As result of that troubled collaboration, Salmon published the first report of *Salmonella* spp. as the cause of hog cholera as the sole author of “The discovery of the germ of swine-plague,” reinforcing the dispute with Smith. However, apart from that scientific dispute, their collaboration had a deep impact and gains on public health. Two of those gains were Jonas Salk’s (1914–1995) production of the polio vaccine and development of a vaccine against typhus, both of which were epoch-making discoveries.

The discovery of *Salmonella* and the subsequent research conducted by Salmon and his colleagues have had a profound impact on public health and food safety. *Salmonella* remains a critical pathogen today, and efforts to control and prevent its transmission continue to be a major focus of public health measures worldwide. His public health efforts made Salmon one of the most prominent US-based advocates, which includes his name as one of the pioneers of the One Health concept.

In 1906, amid bureaucratic disputes in Washington, DC, an offer from the government of Uruguay emerged for him to supervise the founding of the Department of Veterinary Medicine at the University of Montevideo. Salmon accepted the position of director and moved to Montevideo, Uruguay. After 5 years, he returned to the United States and settled in Butte, Montana, where he supervised a plant dedicated to the production of hog cholera serum.

Salmon’s life was cut short when he passed away from pneumonia complicating gastric carcinoma on August 30, 1914, at the age of 64. He was buried in the Rock Creek churchyard in Washington, DC. Nevertheless, his significant contributions to veterinary medicine, human medicine, and public health, including food inspection, private practice, government service, foreign service, leadership in identifying bacterial diseases, and One Health, continue to influence those fields today.
